# Relationship between self-reported weight change, educational status, and health-related quality of life in patients with diabetes in Luxembourg

**DOI:** 10.1186/s12955-015-0348-8

**Published:** 2015-09-18

**Authors:** Anastase Tchicaya, Nathalie Lorentz, Stefaan Demarest, Jean Beissel, Daniel R. Wagner

**Affiliations:** LISER –Luxembourg Institute of Socio-Economic Research, 3 Avenue de la fonte, L-4364 Esch/Alzette, Luxembourg; Scientific Institute of Public Health, Brussels, Belgium; INCCI-Institut National de Chirurgie Cardiaque et de Cardiologie Interventionnelle, Luxembourg, Luxembourg

**Keywords:** Diabetes, Health-related quality of life, WHOQOL-BREF, Weight change, Educational status, Luxembourg

## Abstract

**Background:**

The aim of this study was to assess the relationship between self-reported weight change, socio-economic status, and health-related quality of life (HRQOL) in patients with diabetes, 5 years after they underwent coronary angiography.

**Methods:**

Between 2013 and 2014, 1873 of 4391 patients (319 with diabetes) who underwent coronary angiography between 2008 and 2009 participated in a follow-up study. Three out of four domains of the World Health Organization Quality of Life (WHOQOL)-BREF (physical health, psychological health and social relationships) were surveyed during the follow-up period. To assess the relationship between weight change and HRQOL, generalized linear models were constructed for every dimension of the WHOQOL-BREF, with educational level as a predictor and sex, age, marital status, smoking status, hypertension, cholesterol, ischemic heart disease, acute myocardial infarction, and stable angina pectoris as covariates.

**Results:**

The mean age of the patients was 70 years and almost three-quarters of the patients (72.7 %) were men. During the 12 months preceding the follow-up survey, 22.6 % of the patients reported weight loss, 20 % reported weight gain, and 57.4 % reported no weight change. There were significant differences in the HRQOL scores between patients who reported weight loss and those who reported either weight gain or unchanged weight. The most affected domains were physical and psychological health, with higher scores for patients who reported weight loss (54.7 and 67.2, respectively) than those who reported weight gain (46.3 and 58.5, respectively). The generalized linear model confirmed higher HRQOL scores among patients who reported weight loss and revealed an association between the HRQOL score and education level.

**Conclusion:**

Weight change and education level were associated with HRQOL in patients with diabetes. Self-reported weight loss and no weight change were positively associated with HRQOL in patients with diabetes, while weight gain was negatively associated with HRQOL.

## Background

Diabetes is one of the most important public health issues and the incidence is steadily increasing worldwide. Currently, more than 285 million individuals worldwide are affected by the disease, and according to the International Diabetes Federation, this figure will increase to 438 million by 2030 [1]. According to the American Diabetes Association, diabetes not only causes an economic burden, but also has substantial societal consequences in terms of reduced quality of life and pain and suffering of individuals with diabetes, and affects the family and friends of the individuals [2]. Indeed, health-related quality of life (HRQOL) is influenced by clinical changes that are consequences of treatment and monitoring of the disease [3–5]. Furthermore, diabetes has multiple physical and psychological effects on patients [6].

Overweight and obesity are considered to be the main contributors to the global diabetes epidemic. Most patients have type 2 diabetes, which has been linked to aging of the population and a rapid increase in the number of overweight or obese individuals. Weight loss in patients with diabetes improves glycaemic control and reduces blood pressure, cholesterol levels, and insulin resistance [7, 8]. It has also been associated with a reduction in total mortality and cardiovascular disease plus diabetes mortality [9]. Several studies have described the impact of weight change or body mass index on HRQOL; indeed, weight loss was associated with an improvement in HRQOL while weight gain was associated with a decrease in HRQOL [5, 8, 10–12]. Other studies have stressed the health risks of weight loss in old or very old (>80 years) individuals [13–16].

Numerous studies have demonstrated socio-economic inequalities associated with the incidence and prevalence of diabetes [17–21]. A precarious socio-economic position has been associated with a higher risk of diabetes throughout the developed world [22]. Although there have been few recent studies of the relationship between HRQOL and socio-economic characteristics in patients with diabetes [8, 23–25], several have shown a positive relationship between HRQOL and income, educational level, or professional status [24, 25]. Education level has often been used as a proxy for socio-economic status, but its specific role in relation to HRQOL has not yet been clarified [24]. The aim of this study was to measure the relationship between self-reported weight change, socio-economic status, and HRQOL in diabetes patients 5 years after undergoing coronary angiography.

## Methods

### Participants

Before undergoing coronary angiography, all patients with cardiovascular disease who were admitted to the National Institute of Cardiac Surgery and Interventional Cardiology (INCCI) in Luxembourg between 1 January 2008 and 31 December 2009 were surveyed regarding their education level, profession, nationality, marital status, and presence of risk factors for cardiovascular disease (obesity, smoking, diabetes, hypertension, cholesterol, physical inactivity, and heredity) [26]. In total, 4391 individuals participated in the study. Five years later, during the period between August 2013 and April 2014, all patients were re-contacted by mail and asked to participate in a follow-up study by completing a two-part self-report questionnaire regarding socio-demographic and socio-economic characteristics and risk factors (Part 1), and HRQOL (Part 2). Questions used in the initial survey (2008/2009) were repeated to assess changes in socio-demographic and socio-economic characteristics and risk factors. In total, 1873 patients returned completed questionnaires (participation rate of 42.6 %), of which 319 were diabetes patients. Of these 319 diabetes patients, 77.1 % were diagnosed between 2008 and 2009, 20.4 % were new cases diagnosed after the period between 2008 and 2009, and there was no information on diabetes status between 2008 and 2009 for 2.5 % of the patients. All participants who completed the follow-up survey provided informed consent after receiving a document that described the objectives of the study. The study was approved by the National Research Ethics Committee and the National Commission for Data Protection.

### Self-reported weight change

Self-reported weight change, which has been validated in other studies [27–29], was assessed using the following question: ‘In the past 12 months, did you: lose weight, gain weight, or was your weight stable?’. Based on the responses, a self-reported, three-category weight-change variable was constructed that included patients who reported weight gain, weight loss, and stable weight.

### Socio-demographic and socio-economic variables

Age (defined as a continuous variable), sex, and marital status were used as the demographic variables in the analysis. Marital status was defined as a dichotomous variable: ‘married’ or ‘other’. The educational level served as the socio-economic variable. The educational level was defined as the highest diploma level according to the International Standard Classification of Education and was sub-divided into three levels: primary, secondary, and tertiary education [30].

### Cardiovascular risk factors

Three cardiovascular risk factors were selected for this analysis based on their high prevalence among individuals who suffered from cardiovascular disease and diabetes: high blood pressure, high cholesterol level, and use of tobacco. For blood pressure and cholesterol, dichotomous variables were used: ‘yes’ if the patient reported high blood pressure/high cholesterol levels and ‘no’ if the patient did not report suffering from either of these risk factors. The use of tobacco was assessed using three categories: ‘current smoker,’ ‘ex-smoker,’ and ‘never-smoker.’

### Cardiovascular events

Ischemic heart disease, acute myocardial infarction, and stable angina pectoris were the cardiovascular events that were most frequently observed among patients who underwent coronary angiography between 2008 and 2009. Previous studies have demonstrated the impact of complications from diabetes on the occurrence of these two cardiovascular events [31, 32]. Both events were defined as dichotomous variables: ‘yes’ if the patient had the pathology (or a related health problem) and ‘no’ if the patient did not have the pathology.

### Health-related quality of life

Questions from the following domains of the World Health Organization Quality of Life questionnaire (WHOQOL) were employed: ‘overall quality of life and general health’ (2 items), ‘physical health’ (7 items), ‘psychological health’ (6 items), and ‘social relations’ (8 items). The WHOQOL is a generic instrument that is used to assess quality of life, which is defined as ‘the individual’s perceptions in the context of their culture and value systems, and their personal goals, standards and concerns’ [33]. The WHOQOL-BREF is a shorter version of the original instrument that may be more convenient for use in large research studies or clinical trials. A fourth WHOQOL domain, ‘environment’, was considered less relevant in the context of this study.

Three domains of the WHOQOL-BREF (physical health, psychological health, and social relations) were assessed along with two items representing global perceptions of quality of life and assessment of general health. The mean scores for all items in each domain were used as the domain mean scores. In order to generate scores that were comparable between the mean scores calculated using the WHOQOL-BREF and those used in the WHOQOL-100, the WHOQOL group recommended multiplying the mean scores by a factor of four [34].

### Statistical analyses

Data for diabetes patients were analysed as a function of self-declared weight change (weight loss and weight gain) and, in some cases, as a function of sex. Descriptive analyses of socio-demographic and socio-economic characteristics, as well as health and cardiovascular risk factors were presented in terms of the mean (and standard deviation) and frequency (with percentage). Patients who reported weight loss were compared with those who reported weight gain using chi-square tests for categorical variables and t-tests for continuous variables. Associations among variables predicted to influence HRQOL were assessed either using Pearson or Spearman’s correlation coefficients depending on the categorical or continuous nature of the variables [10, 35]. Least square multiple regression analysis was used to examine the relationship between the HRQOL of patients with diabetes (the physical health and psychological health domains) as outcome variables and weight change status, after adjusting for socio-demographic variables (Model 1), cardiovascular risk behaviour (Model 2), and cardiovascular events (Model 3). This analysis was performed using the general linear model procedure of SAS 9.4 (SAS Institute Inc., U.S.A.).

## Results

Among patients with diabetes (mean age: 70.1, standard deviation: 9.4, men: 72.7 % and women: 27.3 %), 22.6 % reported weight loss in the 12 months preceding the follow-up survey, 20 % reported that they had gained weight, and 57.4 % reported no weight change. The characteristics of the study population according to weight change status are shown in Table 1. Statistically significant differences between groups were observed only for cholesterol levels and ischemic heart disease. Patients with high cholesterol levels accounted for 74.1 % of those who reported weight gain, 55.7 % of those who reported weight loss, and 53.8 % of those who reported no weight change (p < 0.05). In addition, ischemic heart disease was associated with weight change (p < 0.05).

**Table 1:** Characteristics of the study group according to weight change status in the 12 months before the follow up survey

Characteristics	All (n = 319)	Weight loss (n = 72)	Weight gain (n = 64)	No change (n = 183)	*P* value
Age, years, mean (SD)	70.1 (9.4)	69.1 (8.2)	69.9 (10.3)	70.6 (9.6)	0.4864
Sex					
Men	72.7	75.0	65.6	74.6	0.3440
Women	27.3	25.0	34.4	25.4	
Marital status					
Married	74.0	70.8	64.1	78.5	0.0641
Other	26.0	29.2	35.9	21.6	
Education level					
Primary	44.5	45.8	51.6	41.4	0.5608
Secondary	44.2	45.8	39.1	45.3	
Tertiary	11.3	8.3	9.4	13.3	
Hypertension					
Yes	61.5	60.0	69.6	59.4	0.3815
No	38.5	40.0	30.4	40.7	
Cholesterol problems					
Yes	58.0	55.2	74.1	53.8	0.0231
No	42.0	44.8	25.9	46.2	
Smoking status					
Smoker	8.5	14.1	6.4	7.2	0.2067
Ex-smoker	60.6	52.1	52.4	60.2	
Never smoker	30.9	33.8	41.3	32.6	
Ischemic heart disease					
Yes	9.4	4.2	4.7	13.3	0.0286
No	90.6	95.8	95.3	86.7	
Acute Myocardial Infarction					
Yes	16.3	19.4	14.1	16.0	0.6837
No	83.7	80.6	85.9	84.0	
Angina pectoris					
Yes	49.5	51.4	51.6	48.1	0.8351
No	50.5	48.6	48.4	51.9	

Source: Monitoring and DYNamics of health status through the Risk Factors for Cardiovascular disease (MDYNRFC) Survey, 2013/2014

The associations between selected domains of the WHOQOL-BREF and weight change status in patients with diabetes are shown in Table 2. For men, the mean scores for the physical and psychological health domains, and for the overall perception of quality of life were significantly associated with weight change status. In contrast, the mean scores for the social relations domain were independent of weight change status. For women, only the physical health domain was associated with weight change status.

**Table 2:** Health-related quality of life (% of ‘good or very good’ quality) of the study population according to weight change status

Domains of WHOQOL-Bref	All (n = 319)	Lost weight (n = 72)	Gained weight (n = 64)	No change (n = 183)	*P* value
Physical health, mean (SD)	53.2 (17.9)	54.7 (16.7)	46.3 (18.7)	55.4 (17.4)	0.0025
Psychological, mean (SD)	65.3 (17.7)	67.2 (18.7)	58.5 (16.6)	67.4 (16.9)	0.0021
Social relationship, mean (SD)	65.6 (21.9)	70.0 (30.2)	65.6 (14.6)	63.9 (20.7)	0.7675
Overall perception of quality of life, %	59.3	55.6	48.4	65.4	0.0434
Overall perception of general health, %	47.7	53.7	34.9	50.6	0.0591
Overall perception of satisfaction of life, mean (SD)	7.0 (2.1)	7.1 (2.0)	6.3 (2.4)	7.2 (1.9)	0.0112

Source: Monitoring and DYNamics of health status through the Risk Factors for Cardiovascular disease (MDYNRFC) Survey, 2013/2014

The results of the application of three models for assessing the associations between scores on the WHOQOL domain of physical health and weight change status in patients with diabetes are shown in Table 3. In Model 1, the association between score and weight change status was adjusted for socio-demographic variables. In Model 2, variables related to cardiovascular risk behaviour were added to the model. Finally, in Model 3, cardiovascular events were added. Model 1 demonstrated that the mean scores for patients who reported weight loss (+8.4) or no weight change (+9.1) were significantly higher compared to the mean scores for physical health for patients who reported weight gain after controlling for age, sex, marital status, and education level. The addition of variables related to cardiovascular risk behaviour (Model 2) and cardiovascular events (Model 3) did not alter this association. The education level of the patients was independently associated with weight loss status.

**Table 3:** Association between the physical health domain of WHOQOL and weight change status, demographic factors, education level, cardiovascular risk factors and cardiovascular events

Characteristics	Model 1	Model 2	Model 3
Weight change status (ref: gained weight)			
Lost weight	**8.250**	**8.135**	**7.772**
	0.0077	0.0191	0.0269
No change	**7.857**	**7.109**	**6.789**
	0.0032	0.0157	0.0226
Age, years	0.074	0.113	0.116
	0.5148	0.3843	0.3734
Gender (ref : women)			
Men	2.153	3.784	3.186
	0.3785	0.2259	0.3170
Marital Status (ref : other)			
Married	3.753	4.407	4.487
	0.1406	0.1232	0.1208
Education level (ref: primary)			
Secondary	3.249	3.039	3.466
	0.1412	0.2135	0.1654
Tertiary	**8.308**	**10.056**	**10.551**
	0.0159	0.0078	0.0056
Smoking status (ref: current)			
No, ex-smokers		0.966	0.823
		0.8063	0.8351
No, never		4.054	3.729
		0.3451	0.3932
Hypertension (ref: yes)			
No		1.831	1.597
		0.4868	0.5461
Cholesterol problems (ref: yes)			
No		0.689	0.844
		0.7905	0.7466
Ischemic Heart Disease (ref: yes)			
No			-4.653
			0.2102
Acute Myocardial Infarction (ref: yes)			
No			-2821
			0.5321
Angina pectoris (ref : yes)			
No			-0.854
			0.7666

Figures in bold were significant statistically at the 5 % threshold

Source: Monitoring and DYNamics of health status through the Risk Factors for Cardiovascular disease (MDYNRFC) Survey, 2013/2014

The results of the application of the three models to assess associations between scores on the WHOQOL psychological health domain and weight change status in patients with diabetes are shown in Table 4. As was the case for physical health, the mean scores for the psychological health of patients who reported either weight loss or no weight change were significantly higher (respectively, + 8.6 and + 7.6) than those of patients who reported weight gain (Model 1). The addition of variables related to cardiovascular risk behaviour (Model 2) and to cardiovascular events (Model 3) did not alter this association. The mean scores for psychological health were independently associated with patient age whereas education level and physical health were associated with psychological health.

**Table 4:** Association between the psychological health domain of WHOQOL and weight change status, demographic factors, education level, cardiovascular risk factors and cardiovascular events

Characteristics	Model 1	Model 2	Model 3
Weight change (ref: gained)			
Lost weight	**8.618**	**7.311**	**7.411**
	0.0045	0.0268	0.0261
No change	**7.565**	**7.580**	**7.371**
	0.0037	0.0068	0.0095
Age, years	**0.288**	**0.327**	**0.332**
	0.0106	0.0087	0.0082
Gender (ref : women)			
Men	2.791	1.985	1.813
	0.2444	0.5026	0.5489
Marital Status (ref :other)			
Married	2.957	4.174	4.182
	0.2375	0.1270	0.1325
Education level (ref: primary)			
Secondary	4.126	2.707	2.765
	0.0578	0.2459	0.2476
Tertiary	**7.835**	**7.634**	**7.526**
	0.0204	0.0333	0.0376
Smoking status (ref: current)			
Ex-smokers		-1.483	-1.501
		0.6928	0.6910
Never-smokers		1.696	1.217
		0.6771	0.7694
Hypertension (ref: yes)			
No		0.536	0.675
		0.8314	0.7902
Cholesterol problems (ref: yes)			
No		3.342	3.158
		0.1778	0.2067
Ischemic Heart Disease (ref: yes)			
No			1.306
			0.7121
Acute Myocardial Infarction (ref: yes)			
No			-2.603
			0.5509
Angina pectoris (ref : yes)			
No			-0.437
			0.8739

Figures in bold were significant statistically at the 5 % threshold

Source: Monitoring and DYNamics of health status through the Risk Factors for Cardiovascular disease (MDYNRFC) Survey, 2013/2014

## Discussion

In the present study, the associations between weight change, socio-economic characteristics, and quality of life in patients with diabetes were investigated. No association was found between weight change and the socio-demographic or socio-economic characteristics of patients with diabetes. However, high levels of cholesterol and a history of ischemic heart disease were associated with weight change.

The distribution of the quality of life scores for the physical and psychological health domains revealed different statistically significant associations with the socio-demographic and socio-economic characteristics of patients with diabetes. Age was considered to be an important variable when evaluating HRQOL in patients with diabetes [36]. It was previously associated with physical functioning and certain aspects of well being [36]. In our study, age was significantly associated with quality of life in patients with diabetes, but its effect was limited to the psychological health domain and was not linear. Our analysis demonstrated that men had higher scores than women for both indicators of quality of life. Although previous studies have provided evidence that HRQOL is lower in females than in males [37], our study did not show any significant association between gender and quality of life after adjustment for other covariates.

The association between education level and quality of life scores for the physical and psychological health domains was suggestive of a social gradient, as the scores increased with education level. In contrast to the findings of Chaturvedi et al. [38], our data did not support an inverse relationship between socio-economic status and HRQOL in patients with diabetes. With respect to education level as a proxy for socio-economic status, the results showed that the scores were higher for those with tertiary education than for those with primary education. This could be interpreted as one positive effect of education in that it is an important resource that enables individuals to better comprehend, adapt, and adhere to complex new treatments [39] and recommendations that could improve lifestyle. Those with lower education levels have more problems related to self-management of diabetes on a daily basis [39].

Previous studies have shown that social support was directly beneficial for the well-being of patients with diabetes [40, 41]. Jacobson et al. [41] showed that unmarried or divorced patients with diabetes had lower HRQOL scores than married patients. In our study, the results were similar for the global perception of quality of life. In contrast, no statistically significant associations were identified between marital status and the domains of physical and psychological health.

Our study did reveal an association between HRQOL and weight change, specifically for the physical and psychological health domains. The relationship was also statistically significant for global perceptions of quality of life (p < 0.05) and perceptions of satisfaction in life (p < 0.05). In some studies, obesity was associated with decreased HRQOL in patients with diabetes [10, 42]. Patients who reported weight loss experienced improvements in the physical health domain, self-esteem, and global HRQOL compared to patients who reported weight gain [3, 42]. Our results demonstrated a significant association between weight change and HRQOL, particularly with respect to physical health. Further, they confirm the results of other studies in that patients who reported weight gain in the 12 months preceding the survey had a lower HRQOL score.

Differences in HRQOL scores between patients with diabetes who lost weight and those who gained weight could be explained by the fact that overweight (or obesity) has been associated with several psychological problems such as depression and anxiety [43]. In a study of the impact of weight loss on depression in patients with type 2 diabetes, Grandy et al. [42] found (after correction) that patients who lost weight showed a significant improvement in the depression score (p < 0.05) and had a two to three times higher probability of an improvement in the severity of depression compared to patients who gained weight. Our results, which showed that weight loss was associated with an improvement in the quality of life of patients with diabetes, support the findings of the multi-national Diabetes Attitudes, Wishes, and Needs study, which showed that one of the highest priorities of patients with diabetes was to avoid weight gain [44].

Finally, the use of tobacco has often been associated with weak scores for the different dimensions of HRQOL, particularly in patients with diabetes [45]. However, this could not be confirmed in our study.

### Limitations and strengths of the study

Our study of patients who were admitted to the INCCI is not representative of all patients with cardiovascular disease in Luxembourg. Thus, the small sample size was a limitation of our study. In addition, since no information on quality of life was available at baseline, it was not possible to study the evolution of quality of life among patients. However, our study was based on relatively long-term follow-up of patients after coronary treatment compared to numerous other studies regarding HRQOL [46]. To our knowledge, this is the first study of this type in Luxembourg. Self-reported weight change reflects the perception that individuals have of their own weight. As HRQOL is also based on patient perception of well-being, we decided it was appropriate to use self-reported weight change in our study. The validity of this measurement was also demonstrated by several previous studies [27–29]. In a study that compared self-reported weight gain and measured weight gain in women who were using contraception, self-reported weight gain was determined to be a reasonable proxy for true weight gain [27]. We considered that this observation was also applicable for weight loss. In a study of 4760 adolescents and young adults, it was observed that weight change based on a series of self-reported body weight was a valid estimate with minimal error [29].

It is possible that the situation is different for elderly patients. Indeed, aging causes inevitable physiological changes in body composition and fat distribution, which presents a challenge in clinical practice [13]. Thus, with age, even if weight remains stable, the proportion of body fat can increase with a loss in height, which is common in the elderly and is typically due to the narrowing of the intervertebral disc space, osteoporotic vertebral compression and kyphosis [13]. Self-reported weight and height might therefore introduce measurement errors when individuals are classified into relative weight categories. In our study, we opted to use a subjective measure of weight change, which was based on the perception of patients with cardiovascular disease and diabetes of the evolution of their weight during a given time period. Self-reported weight change is thought to be relatively independent of potential biases associated with self-reported weight or height measurements.

Overweight and obesity are considered to be major risk factors for both cardiovascular disease and diabetes, and weight loss is recommended given its potential beneficial effects on health outcomes, well-being, and HRQOL [13, 28, 47, 48]. Whether weight loss should be recommended in the elderly, especially among individuals who are ≥80 years old, remains controversial owing to concerns surrounding the difficulty of behaviour changes with advancing age, age-related loss of skeletal muscle and bone, the feasibility of long-term weight maintenance, and related health consequences [13, 14, 16]. Weight loss, whether intentional or unintentional, may potentially have adverse effects on the health of older adults if it is not combined with regular physical activity [14, 15]. However, other studies have found that muscle quality and physical function in elderly individuals improved with weight loss [15]. Given the advanced age of some of the patients in our study, moderate weight loss could be considered beneficial to health and HRQOL.

Health-related quality of life has become increasingly important in clinical research over the last 15 years [3, 25, 49]. The results of this study will further our knowledge of the impact of changes in risk factors and socio-economic factors on quality of life in patients suffering from cardiovascular disease and diabetes 5 years after coronary angiography.

## Conclusion

Our follow-up study of patients with cardiovascular disease and diabetes has demonstrated significant relationships between weight change, education level and HRQOL. These results highlight the importance of promoting activities that will improve self-management of diabetes such as weight management, lifestyle improvement, and adherence to drug treatment. Measurement of HRQOL could therefore help clinicians to adapt treatment approaches to individual patient needs. In addition, our results can serve as a basis for future studies that aim to assess the evolution of HRQOL.
